# Efficacy of transcutaneous perineal electrostimulation versus intracavitary anal electrostimulation in the treatment of urinary incontinence after a radical prostatectomy: randomized controlled trial study protocol

**DOI:** 10.1186/s12894-020-00718-y

**Published:** 2021-01-28

**Authors:** R. Pané-Alemany, I. Ramírez-García, A. Carralero-Martínez, L. Blanco-Ratto, S. Kauffmann, E. Sánchez

**Affiliations:** 1 Rehabilitación Abdomino-Pelviana (RAPbarcelona SL), Barcelona, Spain; 2 Servicio de Fisioterapia, Instituto Médico Tecnológico SL, Barcelona, Spain; 3 Blanquerna School of Health Science-Universitat Ramon Llull, Barcelona, Spain; 4 Fundació Universitària del Bages (FUB), Barcelona, Spain; 5 Universidad Internacional de Catalunya (UIC), Barcelona, Spain; 6 Servicio de Fisioterapia, Womens Salud Y Bienestar de La Mujer SL, Barcelona, Spain

**Keywords:** Male urinary incontinence, Postprostatectomy incontinence, Surface electrodes electrostimulation, Intra-anal probe electrostimulation, Randomized controlled trial

## Abstract

**Background:**

Radical prostatectomy is the gold standard treatment for men with localized prostate cancer. This technique is associated with post-operative urinary incontinence. Pelvic floor physiotherapy is a conservative, painless and economical treatment for this specific situation. Kegel exercises and perineal electrostimulation are common techniques to train pelvic floor muscles. The perineal electrostimulation can be applied to the patient with surface electrodes or by an intra-cavitary anal probe. This study proposes that transcutaneous perineal electrostimulation is as effective as intra-cavitary electrostimulation in reducing urinary incontinence secondary to radical prostatectomy. The main objective is to compare the efficacy of the treatment with transcutaneous perineal electrostimulation versus the same intra-cavitary treatment to reduce the magnitude of urinary incontinence after radical prostatectomy, and the impact on the quality of life.

**Methods:**

This single-blind equivalence randomized controlled trial will include 70 man who suffer urinary incontinence post radical prostatectomy. Participants will be randomized into surface electrodes group and intra-anal probe group. The groups will receive treatment for 10 consecutive weeks. Outcomes include changes in the 24-h Pad Test, and ICIQ-SF, SF-12 and I-QoL questionnaires. Clinical data will be collected at baseline, 6 and 10 weeks after the first session, and 6 months after the end of treatment.

**Discussion:**

The results will allow us to prescribe the most beneficial perineal electrostimulation technique in the treatment of urinary incontinence derived from radical prostatectomy.

**Trial registration:**

ClinicalTrials.gov Identifier: NCT03587402. 27/06/2018

## Background

Radical prostatectomy (RP) is the gold standard treatment for men with localized prostate cancer and prostatic hyperplasia. Improvements in anatomical knowledge and surgical techniques have significantly reduced post-intervention morbidity [1]. However, RP is associated with post-operative urinary incontinence (UI), that can persist for two years or longer and is associated to significant reductions in overall health-related quality of life (QoL) [2–5].

Urinary continence in men depends on the contributions from smooth muscle of the urethra and urethral constriction generated by contraction of three striated muscles: the striated urethral sphincter (SUS); puborectalis/pubovisceralis and bulbocavernosus [6]. RP inherently removes the prostatic segment of the urethra, and its smooth muscle (called the internal sphincter), and may remove or damage the SUS muscle or its innervation [7]. Continence recovery after prostatectomy is likely to require enhanced function of SUS (and other striated muscles) to compensate for the reduced smooth muscle [6, 7].

Pelvic floor muscle training (PFMT) is the most common non-invasive intervention for UI derived from a radical prostatectomy. Available published evidence demonstrates that PFMT with muscular electrostimulation (ES) has a significant positive impact on the early recovery of urinary continence after this intervention [8–10].

The perineal ES can be applied to the patient with surface electrodes or by an intra-cavitary anal probe [11]. Up to now, the most described method of application in the literature has been intra-anal [10, 12]. Each technique stimulates different anatomical points and remains unknown if both have the same effectiveness or one of them has more effect. Intra-cavitary application can be uncomfortable or annoying for patient; nevertheless, perineal surface ES could be a simple therapeutic modality, easy to apply and equal or more effective than intra-cavitary one.

The study hypothesizes that perineal surface ES is as effective as intra-cavitary ES in the reduction of UI secondary to radical prostatectomy. We aim to compare the efficacy of both techniques in reducing the magnitude of UI secondary to radical prostatectomy, and to evaluate its impact on the patients’ quality of life. Specifically we plan to estimate the amount (grams) of urine lost in 24 h to evaluate the severity of UI secondary to radical prostatectomy, and to assess the quality of life of the patients participating in the study.

## Methods

### Study design

This manuscript describes a research protocol for a single blind, equivalence randomized controlled clinical trial. Equivalence trials aim to determine whether one intervention is therapeutically similar to another; equivalence is defined as the treatment effect being between − Δ and + Δ, Δ meaning the margin of equivalence. If the confidence interval of the outcomes difference between the two studied groups falls within the predetermined margin of equivalence (− Δ to + Δ), the two types of techniques can be considered equivalent. Participants will be randomly allocated into surface electrodes group (intervention group, IG) and intra-anal probe group (control group, CG). The investigator performing the statistical analyses will be blinded.

### Study locations

This trial will take place at RAPbarcelona pelvic floor specialized physiotherapy centre in Barcelona, and at the pelvic floor rehabilitation unit of the Institut Mèdic Tecnologic of Barcelona.

### Study population

Patients who consult in one of the two participating centres for UI derived from a radical prostatectomy surgical intervention will be invited to participate during his first physiotherapy visit.

To be eligible, participants must meet the following criteria. Inclusion criteria: having undergone radical prostatectomy, presenting involuntary urine losses after radical prostatectomy intervention (UI grade I, II or III), do not exceeding the year since the surgical intervention, and accepting to participate in the study granting signed informed consent. Exclusion criteria: following a pharmacological treatment for UI, presenting anatomical malformations of the pelvic floor musculature, carrying a pacemaker, presenting anal fistulas, suffering serious psyche disorders, having a history of lower urinary tract infections, requiring radiotherapy as adjuvant treatment, being diagnosed with urethral stricture after surgery, presenting pelvic floor denervation, and suffering neuromuscular diseases.

### Investigations

Patients who agree to participate in the study will be cited by telephone and receive ES therapy once a week. At baseline, participants will undergo an initial assessment where data on age, date of surgery, surgical intervention technique and days of catheterization will be collected. The Oxford test (to measure the pelvic floor muscular strength), the 24-h Pad Test (in order to quantify the involuntary loss of urine), the UI questionnaire ICIQ-SF, and the SF-12 questionnaire and the specific I-QoL test (to assess their quality of life) will be evaluated at first visit.

A total of ten treatment physiotherapy sessions will be held on a weekly basis (Table 1).

**Table 1:** Treatment sessions

SESSION 1	Collection and recording of baseline data (age, intervention date and intervention technique), Oxford measurement and tests (24-h Pad Test, ICIQ-SF, SF-12, I-QoL) Delivery of the compliance form ES (10 min at 30 Hz and 5 min at 50 Hz) Explanation of the routine for home pelvic floor exercises (10 contractions maintained and 10 fast contractions to be performed three times a day)
SESSION 2	Session protocol: Registration of possible discomfort or adverse effects perceived by the patient Review of the domiciliary exercises routine and record of compliance ES (10 min at 30 Hz and 5 min at 50 Hz)
SESSION 3–5	Session protocol (as described in session 2)
SESSION 6	Collection of 24-h Pad Test data Oxford measurement Record of patient satisfaction with the treatment Session protocol (as described in session 2)
SESSION 7–9	Session protocol (as described in session 2)
SESSION 10	Session protocol (as described in session 2) Oxford measurement Collection of 24-h Pad Test data and completion of ICIQ-SF, SF-12 and I-QoL tests Record of patient satisfaction with the treatment
SESSION 11 (6 months after the end of treatment)	Collection of the 24-h Pad Test, compliance and satisfaction data

According to the allocation group, the ES technique will be applied using the Neurotrac Pelvitone® muscular electrostimulator, together with two round surface electrodes of 32 mm or an Analys Plus® anal stimulation probe of 140 mm.

In both groups, participants will be placed comfortably in a supine position with a pillow under their heads, without pants or underwear, and with the legs bent over two leg supports.

Participants in the IG will receive the treatment through a 32-mm surface electrode attached just above the base of the penis and below the pubis, and another 32-mm surface electrode placed on the perineum (area between the end of the testicles and the anal sphincter).

Patients in the CG will receive the same treatment by means of an anal stimulation probe, which will be placed inside the rectal cavity.

#### *Description of the treatment session* (same for both groups)

15 min of perineal ES (with surface electrodes or intra-anal probe according to the allocation to IG or CG, respectively), composed of 10 min of direct current at 30 Hz frequency and a pulse width of 250 microseconds, to train the tonic fibres, and 5 min of alternating current at 50 Hz frequency and a pulse width of 250 microseconds, to train the phasic fibres.

Additionally, Kegel active exercises are going to be performed under the supervision and correction of the physiotherapist in each of the treatment sessions and also carried out at home. This regimen will consist of ten slow and maintained contractions of the perineal musculature (8–10 s) and ten fast contractions (3 s) to be done three times a day (twice in a supine position and once in a sitting or standing position) during the ten weeks the whole treatment lasts.

In each session, treatment adherence and possible adverse effects of the therapy will be identified and recorded in a database designed for the project.

The same treatment protocol will be followed in all sessions. In the sixth session the results of a second 24-h Pad test will be registered, the Oxford test delivered and the satisfaction with the treatment of each patient recorded. In the tenth session, the same tests as in the first session will be re-evaluated.

The indications for the 24-h Pad test will be given in the session previous to the scheduled for the patient to do it at home and bring the results on the following treatment session, excepting for that of session 1 that will be delivered at the time of acceptance in the study.

Six months after the end of the treatment, patients will be contacted by telephone for a final assessment with the purpose of checking whether the possible benefits of the therapy persist.

### Outcome measures

Participants will complete four study assessments: baseline, 6 and 10 weeks after first session, and 6 months after the end of treatment.

#### Primary outcome

##### Magnitude of urinary incontinence

According to the grams of urine collected in a pad with the 24-h Pad Test. This is a quantitative variable (expressed in grams) evaluated in the first, sixth, tenth and eleventh sessions of the study. The Pad test consists of weighing a clean pad in grams (by the same patient), then placing it and weighing it again after an established time (24 h). In case of needing an extra pad before the 24 h are completed, the procedure would be repeated until 24 h were reached. The increase in weight tells us about the severity of incontinence. The values for mild UI are from 1.3 to 20 g, moderate from 21 to 74 g and severe 75 g or more [13, 14].

#### Secondary outcomes

##### Severity of the urinary incontinence

Assessed with the score obtained in the questionnaire ICIQ-SF, a tool validated and translated into Spanish, specific to the study of UI, used in the basic evaluation of UI from the patient's perspective [15–22]. This is a quantitative variable evaluated in the first, tenth and eleventh sessions of the study.

##### Quality of life related to health

Measured with the SF-12 Quality of Life Scale and the I-QoL questionnaire. The SF-12 health questionnaire is a generic questionnaire. We will use the Spanish adaptation done by Alonso et al. [16, 17] of the SF-12 Health Survey [18, 19]. The SF-12 is a reduced version of the SF-36 Health Questionnaire designed for cases in which this is too long. The SF-12 is answered in an average of ≤ 2 min and the SF-36 needs between 5 and 10 min. It consists of 12 items from the 8 dimensions of the SF-36 physical function, social function, physical role, emotional role, mental health, vitality, body pain and general health status. Higher score means better quality of life. The I-QoL is a specific questionnaire, with only 22 items, that assesses different domains such as avoidance of behaviours, psychosocial impact and feeling of being ashamed. The questionnaire is assessed according to its different scales or globally, so that the higher the scores obtained, the better the quality of life shown [20, 21].

##### Adverse effects

Recorded in each of the treatment sessions through the patient references about his status and evolution.

##### Adherence to treatment

Assessed in each of the treatment sessions, collected through a compliance form designed for the project.

##### Satisfaction of the participants with the treatment

Registered in the sixth and tenth sessions with a scale from 1 to 10 (being 1, not satisfied and 10, very satisfied).

### Schedule

**Table:** 

	Recruitment	Session 1	Sessions 2–5	Session 6	Sessions 7–9	Session 10	6 months post treatment
*Recruitment*							
Selection screening	*X*						
Informed consent	*X*						
Collection of baseline data		*X*					
*Interventions*							
Intervention CG	*X*	*X*	*X*	*X*	*X*	*X*	*X*
Intervention IG	*X*	*X*	*X*	*X*	*X*	*X*	*X*
*Evaluations*							
24-h Pad Test		*X*		*X*		*X*	*X*
Oxford		*X*		*X*		*X*	
ICIQ-SF		*X*				*X*	
SF-12		*X*				*X*	
I-QoL		*X*				*X*	
Compliance form		*X*	*X*	*X*	*X*	*X*	*X*
Recording of adverse effects			*X*	*X*	*X*	*X*	*X*
Treatment satisfaction						*X*	*X*

The different achedule phases are shown in italics

### Sample size

To estimate the sample size, we use the randomisation and online databases for clinical trials program of the online computer software Sealed Envelope Ltd. 2001–2015 (https://www.sealedenvelope.com/power/continuous-equivalence/).

For this estimate, alpha values of 5% and beta of 20% (power of 80%) were taken into account. Based on data published in the literature [10, 12], and considering an expected difference in the 24-h Pad Test between the two studied groups of 22 g, 32 patients are needed in each arm of the study. The interpretation is as follows: “if there is truly no difference between the control (ES by intra-anal probe) and intervention (ES by surface electrodes) treatment, then 64 patients are required to be 80% sure that the limits of a two-sided 90% confidence interval will exclude a difference in means of more than 22 g".

Assuming that there could be losses to follow-up, the number of individuals to be recruited will be increased by 10% until reaching 70 patients (35 patients per group).

### Selection of the sample

The selection of the sample will be done by sampling of consecutive cases, from RAPbarcelona clinic and Institut Médic Tecnológic in Barcelona. In urologists or physiotherapists appointments, patients who underwent radical prostatectomy and presented with UI will be referred to the principal investigator. The protocol will be clearly explained to each of the interested patients who meet all the selection criteria and he will be asked to sign the informed consent if accept participation. After the signature the patient will be allocated to one of the two study groups.

### Random allocation of groups

A consecutive inclusion will be done until the desired sample size is reached. The generation of the random sequence will be carried out by the principal investigator using the statistical software Epidat. The randomization list will be delivered to the clinics reception desk and patients will be allocated by telephone at a 1:1 ratio (assignment by third parties).

Each participant will be assigned an identification number created for the study and linked to his treatment group. This number will allow the patient for beginning the treatment sessions. Two physiotherapists previously trained for the application of the study protocol will be in charge of the sessions.

### Collection, management and data analysis

Data will be collected in a specific database for this study by the physiotherapists in charge of the application of the technique and sent to a data analyst unfamiliar with the techniques and study groups. The SPSS 24.0 software will be used.

First, a descriptive analysis of the characteristics of the patients included in both study groups, as well as the outcome variables will be carried out. To do this, absolute and relative frequencies (percentages) will be estimated for qualitative variables, and mean or median and standard deviation or range, respectively, depending on the normality of the distribution, for quantitative variables.

In addition, different associations between diverse variables will be analysed.

To check for the equivalence of the study treatments efficacy, intention to treat (ITT) and by protocol (PP) analysis will be performed.

The comparison of results will be done by estimating the differences in a timely manner and with their corresponding 90% confidence intervals (90% CI). Additionally, the adjusted differences will be calculated, following the indications of the CONSORT document [23].

In all cases, the level of statistical significance established will be the usual (5%); Therefore, statistically significant differences will be considered when p values are less than 0.05.

If adverse effects would occur, the physiotherapist together with the principal investigator will decide the continuity of the participant in the study, having his safety and health as a priority.

## Discussion

The result of this study will allow us to prescribe the most beneficial ES technique in the treatment of UI derived from radical prostatectomy. If the surface technique is proven equivalent, it would be more comfortable for the patient to receive the treatment.

## Data Availability

Not applicable.

## Abbreviations

RP: Radical prostatectomy
UI: Urinary incontinence
PFMT: Pelvic floor muscle training
SUS: Striated urethral sphincter
ES: Electro stimulation
QoL: Quality of life
ICIQ-SF: International Consultation on Incontinence Questionnaire Short-Form
SF-12: Health Questionnaire Short Form 12
I-QoL: The Incontinence Quality of Life Questionnaire
IG: Intervention group
CG: Control group
